# Evaluating and Managing the Microbial Contamination of Eye Drops: A Two-Phase Hospital-Based Study

**DOI:** 10.3390/pharmaceutics16070933

**Published:** 2024-07-12

**Authors:** Léa Roquefeuil, Katia Iskandar, Christine Roques, Loïc Marchin, Mylène Guittard, Hélène Poupet, Marie-Laure Brandely-Piat, Marion Jobard

**Affiliations:** 1 Unité de Préparations Stériles Ophtalmologiques et Oncologiques, GHU AP-HP Centre-Université de Paris Cité—Site Cochin, 27 Rue du Faubourg Saint-Jacques, 75014 Paris, France; lea.roquefeuil@aphp.fr (L.R.); marie-laure.brandely-piat@aphp.fr (M.-L.B.-P.); marion.jobard@aphp.fr (M.J.); 2 Department of Pharmacy, School of Pharmacy, Lebanese International University, Beirut 1105, Lebanon; 3 Institut National de Santé Publique, d’Épidémiologie Clinique et de Toxicologie-Liban (INSPECT-LB), Beirut 1103, Lebanon; 4 Laboratoire de Génie Chimique, CNRS, INPT, UPS, Faculté de Pharmacie, Université de Toulouse, 31062 Toulouse, France; christine.roques@univ-tlse3.fr; 5 FONDEREPHAR, Faculté de Pharmacie, 31062 Toulouse, France; 6 Pylote SAS, 22 Avenue de la Mouyssaguèse, 31280 Drémil-Lafage, France; loic.marchin@pylote.com (L.M.); mguittard@pylote.com (M.G.); 7 Laboratoire de Bactériologie, GHU AP-HP Centre-Université de Paris Cité—Site Cochin, 27 Rue du Faubourg Saint-Jacques, 75014 Paris, France; helene.poupet@aphp.fr

**Keywords:** eye drops, dropper tip, cap, microbial contamination, mineral oxide, ceramic, antimicrobial surface

## Abstract

The microbial contamination of eye drop tips and caps varies between 7.7% and 100%. In seeking patient protection and continuous improvement, the Pharmacy Department in the Sterile Ophthalmological and Oncological Preparations Unit at Cochin Hospital AP-HP, Paris, France, conducted a two-phase study to compare the antimicrobial efficiency and practical use of standard packaging and a marketed eye drop container incorporating a self-decontaminating antimicrobial green technology by Pylote SAS at the tip and cap sites. The first phase was conducted in situ to identify the microbial contaminants of eye drops used in the hospital and community settings. A total of 110 eye drops were included for testing. *Staphylococcus species* were the most prevalent bacteria. *Candida parapsilosis* was detected in only one residual content sample and, at the same time, on the cap and tip. The second phase was performed in vitro, according to JIS Z2801. Reductions above one log in *Staphylococcus aureus* and *Pseudomonas aeruginosa* counts were noted in Pylote SAS eye drop packaging after 24 h of contact. The practical tests showed satisfactory results. Pylote SAS antimicrobial mineral oxide technology exhibited promising effects that combined effectiveness, safety, and sustainability to protect the patient by preventing infections due to the contamination of eye drop containers.

## 1. Introduction

Eye health is a state of maximized visual and functional ability that affects society’s overall health, well-being, and quality of life [1]. Ocular diseases have clinical, economic, and humanistic impacts on individual and national bases [1]. According to the World Health Organization (WHO), at least 2.2 billion individuals worldwide have vision impairments [2]. Notably, nearly half of the reported cases are due to cataracts, refractive errors, age-related macular degeneration, glaucoma, or diabetic retinopathy [3]. However, it is important to note that many vision impairment types are preventable, including eye infections [2]. The eye is a complex-structured organ with specialized anatomy and physiology [4,5] and a resilient local immune response [6]. Despite these defenses, ocular infection remains the leading cause of ocular injury and blindness [7]. Such infections can be due to multiple causes, among which the microbial contamination of ophthalmic droppers is significant [8]. These infections are the leading cause of ocular injury and blindness [7]. Specifically, the contamination of eye drops can occur throughout the manufacturing process, along the supply chain, and during use. To address this issue, preservatives are one of the solutions intended to decrease and even eliminate the risk of contamination of the formula [8]. Nevertheless, despite their wide use, these substances do not always have the intended effect [8]. Studies have shown that the microbial contamination rate of in-use therapeutic and diagnostic preserved eye drops varies between 11.7% and 94.46% [9,10,11,12,13,14]. Furthermore, preservatives can have a toxic effect on corneal and conjunctival surfaces [15,16,17]. It is crucial to recognize that the preservative’s effect is limited to the content of the eye drops and cannot prevent the microbial contamination of the dropper tip or cap due to having limited to no contact with these surfaces [8]. Indeed, a thirty-year literature review showed that the microbial contamination rate of preserved and preservative-free eye drops ranges from 2.3% to 73% [8]. Within this range, 7.7% to 100% of cases are due to often-disregarded sources, including the contamination of the dropper tip and cap [8]. Significantly, multiple studies have found an association between contaminated eye drops and eye infections [18,19,20,21,22,23,24,25,26,27,28], including keratitis [23,24,28], corneal ulcers [23,26], and bacterial endophthalmitis [21,27]. In particular, microbial contamination of the dropper tip and cap was associated with corneal injury [20,28] and bacterial keratitis [24]. Thus, the contaminated dropper tip is a potential leading cause of serious ocular infections due to direct contact with the eyes during medication administration [29,30,31,32]. Eye drops provide a noninvasive method for ocular drug delivery intended to treat, diagnose, and prevent eye diseases [33]. As defined by the *European Pharmacopoeia*, eye drops are sterile solutions, emulsions, or suspensions, aqueous or oily, containing one or more active ingredients intended for ocular instillation [34]. Notably, these conventional forms represent 90% of the marketed ophthalmic solutions [33]. They are available as ready-to-use products, either with or without preservative agents [34], and formulated in single-use or multi-use containers [8]. The production of eye drops relies on Good Manufacturing Practices (GMP) guidelines for preparing ophthalmic forms [35]. However, marketed pharmaceutical products do not meet all needs in ophthalmology. Consequently, eye drops are also prepared in hospitals to meet specific patient requirements, including for patients with allergies to preservatives [36]. This production process follows the Good Preparation Practices (GPP) guidelines [37], quality controls, and the methods for preparing sterile products outlined in the *Pharmacopoeia* [34].

The Pharmacy Department at the Assistance Publique des Hôpitaux de Paris (APHP), France, has a dedicated unit for preparing different preservative-free eye drops for hospital and patient use. Specifically, the Sterile Ophthalmological and Oncological Preparations Unit (UPSO2) is a center of excellence that thrives on maintaining sterility throughout the supply chain to ensure patient and medication safety. The manufacturing process follows the recommendations of Good Preparation Practices (GPP) [37] and the *European Pharmacopoeia*, 11th edition, chapter 5.1.1. [34]. It is imperative that eye drops remain sterile throughout the entire manufacturing process and supply chain. Moreover, this sterility should be preserved for the recommended duration of use, from opening to handling by the end user [8,9]. Within this context, UPSO2 evaluated an innovative green technology proposed by Pylote SAS. This technology is incorporated into eye drop tip and cap materials. Notably, the first eye drop container was marketed, as of 2022, collaboratively with the packaging supplier Berry Global. This first-to-market multidose antimicrobial dropper, called Activated RispharmTM, is designed to help prevent microbial eye infections in patients. Importantly, ophthalmic droppers activated by Pylote technology allow a hygienic application, reduce plastic waste, and are fully biocompatible while being certified as non-irritant and non-cytotoxic technology [38].

To evaluate the effectiveness, applicability, and practical use of eye drops activated by Pylote technology, the Pharmacy unit—UPSO2—conducted a two-phase study, as outlined in this article. The first phase assessed the risk of contamination of preservative-free in-use standard eye drops when used by patients and practitioners and identified the possible contaminating microorganisms, taking into account the microbial contamination of eye drops at the cap, tip, and content levels. Subsequently, the second phase was carried out in vitro, comparing the evolution of the artificial microbial contamination of caps and tips between eye drops in standard packaging and those in activated packaging (activated caps and tips, standard bottle) with Pylote technology. Additionally, the study evaluated the practical application and use of the activated packaging. Ultimately, the aim is to validate the effectiveness of green technology in preventing microbial contamination, allowing practical use, and ensuring patient safety.

## 2. Materials and Methods

### 2.1. Study Design

This experimental study encompasses two phases. Phase 1 was conducted in situ and phase 2 in vitro.

#### 2.1.1. Phase 1: The Assessment of the Microbial Contamination of In-Use Eye Drop Content and Dropper Tip and Inner Cap Surfaces

**Figure 1:**
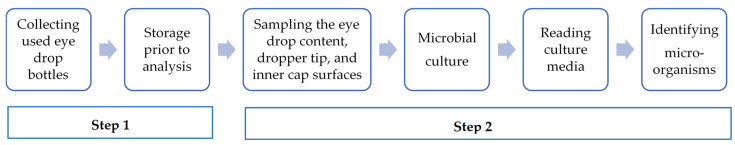
Assessment process of used eye drops’ microbial contamination.

The first phase involves conducting a comprehensive assessment of the level of microbial contamination of compounded eye drops used by patients and healthcare professionals at the Pharmacy Unit—UPSO2. The study was conducted over four months, from 17 January 2023 to 17 May 2023, following the outlined methodology illustrated in Figure 1.

Step 1: Collection and storage of used eye drop bottles

- This study was conducted collaboratively between the Ophthalmology Services and the Pharmacy Unit (PU)—UPSO2.
- The storage temperature of collected eye drops was between +2 and +8 °C for a maximum of 48 h before analysis.
- The information recorded for each eye drop included the collection date, active ingredient, batch number, source, packaging type, storage conditions during use, indication, and duration of treatment.

Step 2: Sampling and culturing the eye drop content and dropper tip and inner cap surfaces to quantitatively and qualitatively determine the contamination level and identify the microbial contaminant.

##### Validating the Assay Conditions

The validation of the sampling and culture methods is a fundamental step to ensuring the reliability of the adapted method. All procedures were conducted within a Class A vertical laminar airflow hood within a Class B cleanroom.

- The selection of microbial contaminants

The dropper tips and inner caps were intentionally contaminated with three microorganisms, as recommended by the *European Pharmacopoeia*, to determine the reliability and validity of the sampling and culturing methods [34]:

1. Gram-positive cocci: *Staphylococcus aureus* (NCTC 10788);
2. Gram-negative bacilli: *Pseudomonas aeruginosa* (NCTC 12924);
3. Fungi: *Candida albicans* (NCPF 3179).

The choice of *S. aureus*, *P. aeruginosa*, and *C. albicans* as the test microorganisms was guided by a thorough review of the literature spanning three decades, which highlighted their frequent isolation as contaminants from eye dropper tips and inner caps across various studies [8]. Moreover, these three microorganisms are recommended for antimicrobial evaluation by the *European Pharmacopoeia* (11th edition) [34] and the ISO 22196:2011 standard [39]. Their inclusion ensures alignment with established guidelines for assessing microbial contamination on pharmaceutical packaging components that come into direct contact with the product during administration.

b. Establishing the number of microorganisms necessary for contamination

Determining the number of microorganisms required for contamination is fundamental to ensuring that the number of microbial contaminants is countable after 24 h of contact with the surfaces. Precultures were prepared from Bioball^®^ (Biomérieux, Marcy-l’Étoile, France), lyophilized hydrosoluble beads containing a determined number of microorganisms (30 ± 3 colony-forming units (CFU) per microorganism).

**Figure 2:**
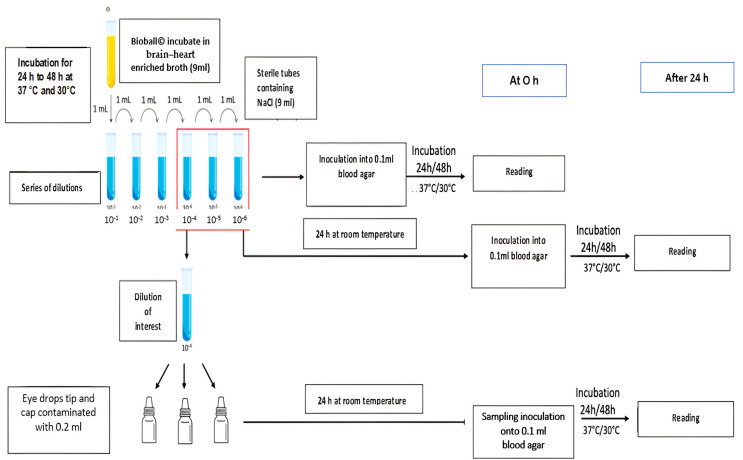
Process for the validation of microbial contamination of eye drop tip and cap.

Bioball^®^ was incubated in 9 mL of brain–heart infusion (BHI) broth (Biomérieux, France) for 24 h at 37 °C for *S. aureus* and *P. aeruginosa* and 48 h at 30 °C for *Candida albicans* (Figure 2, step 1). From the enriched broths (average between 10^8^ CFU and 10^9^ CFU), a ten-fold dilution series was undertaken in sterile 0.9% NaCl (Biomérieux, France), and 100 µL of each dilution was then spread on blood agar (Columbia agar enriched with 5% horse blood, Biomérieux, France) at 0 h and after 24 h at room temperature. This culture medium was selected as it allows the growth of most microorganisms. The colonies were then counted after 24 h of incubation at 37 °C for *S. aureus* and *P. aeruginosa* and after 48 h at 30 °C for *C. albicans* to estimate the microorganism concentration in each dilution tube.

The first agar plate with a countable number of colonies was used to calculate the number of microorganisms in the upstream tubes. The 4th dilution (10^−4^) was chosen as the dilution of interest because it enables counting and detecting microorganisms (no more than 330 CFU/plate) even after 24 h.

c. Microbial contamination of the dropper tip and inner cap of sterile eye drops

**Figure 3:**
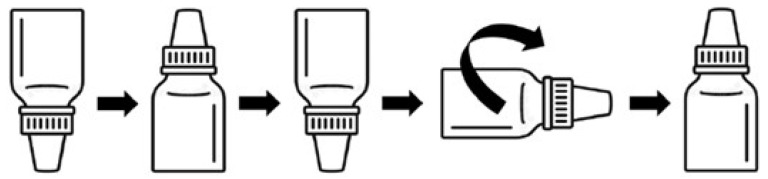
The protocol for the contamination of the eye drop caps and tips.

The protocol for the contamination of the dropper tips and inner caps from the dilution of interest is as follows (Figure 3):

- Volumes of 0.2 mL of the selected dilution, 10^−4^, were deposited on the inner surface of eye drop caps.
- The eye drop bottles were positioned upside down to bring the eye drop tips into contact with the suspension by immersion.
- The eye drop bottles were then repositioned upright to evenly distribute the suspension over the entire surface of the tips and caps.
- Finally, the eye drop bottles were placed horizontally and rolled on the bench.
- The eye drop bottles were incubated upright for 24 h at room temperature.

In parallel, the last 3 dilution tubes, including the dilution of interest, were treated as follows:

- Inoculated (0.1 mL) at H0 onto blood agar plates. These were incubated for 24 h at 37 °C for *S. aureus* and *P. aeruginosa* and for 48 h for *C. albicans*, and they were then analyzed to enumerate the microorganisms (MO) in the dilution of interest.
- Incubated for 24 h at room temperature and then inoculated (0.1 mL) onto blood agar plates. These were in turn incubated for 24 h at 37 °C for *S. aureus* and *P. aeruginosa* and for 48 h for *C. albicans*, and they were then analyzed to enumerate the MOs in the dilution of interest after 24 to 48 h in 0.9% NaCl.

The agar plate corresponding to the dilution of interest was the control.

d. Sampling and culturing the eye drop tips and inner caps

The Bacteriology Department of the Pharmacy Unit—UPSO2—established the microorganism recovery protocol.

- The excess solution in the caps was removed after 24 h of contact with the indicated microorganism.
- The eye drop tips and inner caps were swabbed separately using eSwab^®^ (Deltalab, Spain).
- The agar plates were inoculated by direct inoculation from the swab, which was spread over the entire surface; then, the swab was turned by 90° and spread a second time by turning the Petri dish 90°.
- The blood agar plates were incubated for three days at 37 °C for *S. aureus* and *P. aeruginosa* and at 30 °C for *C. albicans*.

The method was considered validated if the number of MOs collected was equivalent between the 3 vials (in terms of log) for each microorganism in the same run.

##### Collection and Culture of In-Use Specimens

- Identification of Microorganisms by MALDI-TOF-MS

The validated method for the recovery of the 3 test microorganisms was applied to all collected eye drops, maintaining them under controlled storage conditions. The agar plates were inspected after 72 h of incubation to detect microbial growth. Microorganisms isolated on agar plates were identified using the MALDI-TOF microflex^®^ spectrometer (Bruker, France) in collaboration with the Hygiene Department. This technique utilized matrix-assisted laser desorption/ionization time-of-flight mass spectrometry (MALDI-TOF-MS), and the obtained spectra were compared with reference spectra in the database.

b. Evaluation of Residual Content Contamination

When the presence of microorganisms (MOs) was detected on the dropper tip and/or the inner cap, the residual contents of the eye drop bottle were sampled under sterile conditions using a syringe for the sterility test. The sterility test is a microbiological test required by the *European Pharmacopoeia* to verify that sterile products do not contain viable microorganisms [34]. The method used was membrane filtration or STERITEST^®^ (Merck, Lyon, France), which retains microorganisms on a filter with a pore size of 0.45 μm, and all inhibitory compounds are rinsed using the appropriate rinsing solution. Appropriate media (thioglycolate broth and Tryptic Soy Broth—Merck, France), selected based on their ability to promote the growth of anaerobic or aerobic microorganisms, are then used for the transfer of the membrane filters [40]. Evidence of microbial growth is detected by visual observation of turbidity in the culture medium.

#### 2.1.2. Phase 2: Evaluation of the Practical and Antimicrobial Properties of the Pylote SAS Antimicrobial Technology

The second phase evaluated the practical and antimicrobial properties of the Pylote SAS antimicrobial technology applied to the tips and caps of the eye drops.

##### Validation of the Practical Application of Activated Rispharm™

This phase focuses on validating the practical application of the eye drops activated by Pylote SAS technology. The process extends from manufacturing to the end-user experience and provides a comprehensive understanding of the effectiveness and applicability of implementing Pylote SAS technology in healthcare settings.

- Activated Rispharm™ Eye Drops: Practical Evaluation

Integrating the new packaging for eye drops in the UPSO2 production process requires ensuring that it does not cause malfunctions during production or patient use. The packaging evaluation entails producing three fictitious batches of ten placebo eye drops with Activated Rispharm™ packaging by three operators following the applied procedures. Three pharmacists from the unit simulated the use of eye drops by a patient. At each stage, the professionals’ feedback relied on using a form listing critical points.

- The Production of a Batch of Eye Drops in an Isolator: For the production of eye drops in the isolator, the operators had to evaluate the following:
  - The adaptability of the bottleneck to the filling process.
  - The attachment of the dropper tip to the eye drop container.
  - The capping procedure.
- Inspection and Packaging: For the inspection and control of organoleptic characteristics, operators were asked to verify that the bottles were transparent enough to allow the detection of any non-conformities (clarity, absence of particles, color, volume).
- Concerning labeling and packaging, they had to ensure the absence of significant malfunctions on the labeling lines and verify the suitability of the eye drop bottle size for the size of the eye drop boxes used by UPSO2 for secondary packaging.
- Practical Use: A drop was extracted from the eye drops every hour for several consecutive hours to replicate patient use. This administration pattern corresponds to enhanced antibiotic eye drop treatments that must be administered every hour for an average of 2 to 3 days or longer, depending on the severity of the infection.
- Other evaluated aspects included the flexibility of the bottle during manipulation, the consistency of delivered drops, and changes in or issues with the eye drops during use.

##### Validation of the Antimicrobial Properties of Activated Rispharm™

The dropper tip and inner cap surfaces of the standard packaging and Activated^TM^ packaging types were inoculated with a determined number of the pre-selected microorganisms (initial validation step). At 24 h of contact, the tip and inner cap surfaces were cultured as previously described.

The testing groups were as follows:

- Group 1: 3 standard packaging vs. 3 Activated Rispharm™ packaging contaminated with 10^5^ CFU of *S. aureus.*
- Group 2: 3 standard packaging vs. 3 Activated Rispharm™ packaging contaminated with 10^5^ CFU of *P. aeruginosa.*
- Group 3: 3 standard packaging vs. 3 Activated Rispharm™ packaging contaminated with 10^3^ CFU of *C. albicans.*

## 3. Results

### 3.1. Phase 1: Assessment of Microbiological Contamination In-Use Eye Drops

#### 3.1.1. Validation of Sampling and Culture Methods

The results demonstrated that the sampling method is replicable for the three tested microorganisms, *S. aureus*, *P. aeruginosa*, and *C. albicans.* After 24 h of contact time with a deposit of about 10^3^ CFU of *S. aureus* and *P. aeruginosa*, the technique allows the sampling of 10^2^ CFU on the three tested bottles. Similarly, for *C. albicans*, after 24 h of contact time with a deposit of about 10^2^ CFU, the technique allows the sampling of 10^1^ CFU on the three contaminated bottles (Appendix A). These results show that the sampling method for the inserts and inner surfaces of the caps is repeatable for the three tested microorganisms. The sampling method is validated.

#### 3.1.2. Collection and Culture of In-Use Specimens

Over four months, 157 eye drops produced by UPSO2 and used by out-patients and nurses were collected, with 110 eye drops analyzed (hospitalization (65%) versus patients (35%)) and 47 discarded due to a delay in the period between eye drop collection and the analysis of samples.

In-use eye drops collected from the hospital contained antibiotics (90%), including amikacin, piperacillin, vancomycin, and other anti-infective agents (10%), including voriconazole, amphotericin B, and anti-amebic agents. Eye drops collected from patients contained immunosuppressants (87%), notably cyclosporine, antibiotics (2%, piperacillin), and other anti-infective agents (11%), including voriconazole and amphotericin B.

##### Contamination Rate and Characteristics of Contaminated Bottles

Out of 110 collected eye drops, 36 (33%) were contaminated, as follows: 24 (22%) eye drops at the cap and dropper tip levels simultaneously, 9 (8.1%) eye drops solely at the cap level, and 3 (2.7%) solely at the dropper tip level. The microbial loads varied between 1 CFU and 10^2^ CFU per dropper tip/cap.

##### Number of Microorganisms Recovered from Eye Drop Tip and Inner Cap

- The three contaminated eye drop tips and inner caps used during hospitalization had a microbial load of less than 10 CFU.

Out of the 33 contaminated eye drops collected from patients, 20% of cultured dropper tips and inner caps showed a microbial load of around 10^2^ CFU, and 20% had a high level of contamination (>330 CFU). Only one bottle’s residual content showed microbial contamination.

##### Isolated Microorganisms

- The identified microorganisms included commensal germs from the environment and human skin flora. The used eye drops collected from the hospital and patients showed microbial contamination of the eye drop tips and caps with *Staphylococcus epidermidis* and *Stenotrophomonas maltophilia*. All other detected microorganisms originated only from patients using eye drops. The most frequently detected microorganisms from used eye drop tips and caps were Gram-positive bacteria (GPB), such as *Micrococcus luteus* (n = 14) and other GPB considered part of the skin flora, including *Staphylococcus hominis* (n = 5), *Staphylococcus capitis* (n = 4), *Kocuria species* (n = 6), *Staphylococcus aureus* (n = 3), *Staphylococcus haemolyticus* (n = 3), *Staphylococcus warneri* (n = 2), *Staphylococcus saprophyticus* (n = 2), *Staphylococcus auricularis* (n = 1), *Staphylococcus pasteuri* (n = 1), *Corynebacterium species* (n = 2), *Microbacterium aurum* (n = 2), *Aerococcus viridans* (n = 1), and *Dolosigranulum pigrum* (n = 1). Isolated GPB found in the environment included *Bacillus cereus* (n = 2), *Bacillus thuringiensis* (n = 1), *Bacillus licheniformis* (n = 1), *Janibacter hoylei* (n = 2), *Lysinibacillus* spp. (n = 1), and *Streptomyces violaceoruber* (n = 1). Gram-negative bacteria (GNB) contaminants included *Stenotrophomonas maltophilia* (n = 3), *Moraxella osloensis* (n = 2), *Pseudomonas oryzihabitans* (n = 1), *Acinetobacter lwoffii* (n = 1), and *Roseomonas mucosa* (n = 4). Sterility tests showed only one positive result (in-use eye drop bottle collected from a patient) involving the eye drop tip and inner cap contaminated with *Candida parapsilosis* (n = 1). The residual content’s microbial contaminants were *Candida parapsilosis* and *Lysinibacillus* spp. (n = 1).

### 3.2. Phase 2: Evaluation of the Practical and Antimicrobial Properties of Pylote SAS Antimicrobial Technology

#### 3.2.1. Validation of the Practical Application of Activated Rispharm™

- Use of Activated Rispharm™: Minor discrepancies were noted compared to the bottles routinely used. There were no significant differences in the bottleneck size or its adaptability to the filling process. Operators unanimously acknowledged the challenge of inserting the dropper tip. The operators recommended the implementation of a more robust capping procedure to ensure secure bottle closure, with a particular focus on addressing any vacuum occurrence before the final screwing movement.
- Three different operators were involved in evaluating (1) the organoleptic characteristics, (2) labeling, and (3) packaging with Activated Rispharm™ packaging. One operator raised concerns about the space between the cap ring and the bottle body, which may affect the proper closure of the bottles. All operators faced labeling issues because of the width of the Activated Rispharm™ bottles and the parameters of the labeling machine. There were no difficulties with the secondary packaging.
- Three pharmacists were assigned to simulate the administration of compounded eye drops. They all agreed on the flexibility of the Activated Rispharm™ bottles and the dispensing of drops. The Activated Rispharm™ bottles were described as flexible, providing a good grip and allowing the formation of drops with a reproducible volume and controlled administration frequency. The bottle shape during use demonstrated no changes, such as observed deformities, after emptying the bottle.

#### 3.2.2. Validation of the Antimicrobial Properties of Pylote SAS Antimicrobial Technology

The results of the experiment comparing Activated Rispharm™ packaging to standard packaging are presented in Table 1.

The control results at C0 h and after C24 h indicated that the microbial loads of *S. aureus* and *P. aeruginosa* decreased by 2 log and 3 log, respectively. There was no difference in *C. albicans* between C0 and C24h (2.3 × 10^3^ CFU average microbial load).

Assays performed after A24 h with the standard packaging showed the following:

- For *S. aureus*, the standard packaging showed CFU counts higher than the detection limit (3.3 × 10^3^ CFU) 24 h after the cap and insert inoculation. In the same conditions, the agar plates from Pylote packaging showed a countable average of 2.1 × 10^2^ CFU. A difference in the reduction of above 1.2 log in favor of Pylote packaging was observed compared to standard packaging.
- For *P. aeruginosa*, standard packaging also showed CFU counts higher than the detection limit (3.3 × 10^3^ CFU) 24 h after the cap and insert inoculation. Agar plate counts from Pylote^TM^ showed 3.2 × 10^2^ CFU on average, leading to a reduction higher than 1 log.
- For *C. albicans*, both packaging types showed similar microbial loads: 3.0 × 10^2^ CFU for standard packaging and 2.4 × 10^2^ CFU for Pylote^TM^ packaging.

## 4. Discussion

A two-phase study was conducted at the Pharmacy Unit—UPSO2, France. The first phase examined microbial contamination after the use of eye drops compounded by UPSO2, followed by a second phase that evaluated the effectiveness of eye drops incorporating Pylote antimicrobial technology in preventing the contamination of the dropper tip and cap and assessed the practical integration of the innovative packaging into the eye drop compounding process.

In the initial phase, 33% of the tested eye drops used during hospitalization or returned by patients were contaminated by commensal and environmental bacteria and fungi. Those utilized by patients were contaminated (>91%) and exhibited higher microbial loads than the ones collected from the hospital. Consistent with our findings, two studies showed that preservative-free eye drops prepared in the hospital in multidose containers collected from patients had a higher microbial load than those used by healthcare providers [41,42]. The results in this study align with the literature that evaluated preservative-free eye drops used in different settings and showed that microbial contamination varied between 2.3% and 73% [8]. Notably, the microbial contamination of preservative-free multidose eye drops prepared in the hospitals ranged between 8.4% and 28.9% [41,42,43,44]. The microbial contamination of single- and multi-user eye drops is an established risk of infection, documented at a variable rate [14,18,45,46] and in different settings, including hospitals, clinics, and homes, [9,13,14,25,45,47,48,49] whether preserved or preservative-free [9,10,11,12,13,18,28,47,48,49,50,51,52,53,54].

A thirty-year literature review examined the microbial contamination rate of in-use eye drops and found multiple inconsistencies in the literature related to the study design and numerous other contributory factors [8]. The disparity in the reported rate of microbial contamination across different studies may be due to the sampling method, swabbed eye drop sites, the methodology of specimen collection and the transfer of tested samples, differences in the culture technique and media, the testing conditions, and the conducted microbiological assays [8]. In the present study, the microbial load of eye drops collected from the hospital was 8%, compared with 92% in those returned by patients. The hospital’s strict hygiene protocols, the handling of the drug by nurses, and the usually short duration of use, predominantly in the operating room, may have contributed to the limited prevalence of microbial contamination in the hospital setting [14,44,45]. Other potential factors are associated with the type of eye drops collected from in-patients, predominantly comprising antibiotic formulations (90%). While these antimicrobial agents may effectively inhibit the growth of some microorganisms within the eye drop solution, their protective action does not necessarily extend to the external surfaces of the dispensing apparatus, specifically the dropper tip and cap. However, despite the stringent protocols, the microbial contamination of preserved and preservative-free eye drops, including the dropper tip, has been reported in operating rooms [14,44,47,48] and hospital wards with variable durations of use [11,47,52,55,56].

The dropper tip and cap are often overlooked as potential sources of microbial contamination and cross-contamination [8,20,24,28,43,50]. The aforementioned review highlighted the role of the dropper tip and cap in microbial contamination by direct contact with the eye or through the eye drops when they pass through the tip, which ranged between 7.7% and 100% of tested samples [8]. The present study confirmed these findings. These sites (tip and cap) offer a broad surface exposed to commensal and environmental microorganisms that can transmigrate into the content of the eye drops and even come into direct contact with the ocular surface, eyelids, and eyelashes during medication administration, potentially leading to infection and the risk of ocular injury [18,19,20,21,23,57,58,59,60,61,62,63]. Studies have shown that the rate of the eye dropper tip’s direct contact with the ocular surface during drug administration ranges between 18% and 76% in different age groups, predominantly elderly [29,30,31,32,59,64,65,66,67,68,69,70,71,72].

This investigation found that isolated microorganisms belonged to commensal and environmental flora. The human flora is usually nonharmful and even plays a protective role on the ocular surface [18,40,46,48,53,73]. However, the proliferation of this flora, initially harmless, on the tip and the cap can represent a risk to the patient. The risk of ocular injury due to infection is high in individuals undergoing ocular surgery, wearing contact lenses long term, or using topical or systemic corticosteroids or immunosuppressant medications, in addition to those with lid deformities [9,12,14,40,47,48,50,54]. The most frequently isolated bacteria documented as causative agents for ocular injury include *S. aureus*, *S. epidermidis*, and another *Staphylococcus* spp., followed by *P. aeruginosa* [7,74,75]. Similarly, GPB were detected in this study on 90% of the tested dropper tips and caps. Other cultured microorganisms known to cause ocular diseases [76,77,78,79,80,81,82,83,84,85,86,87] include *Corynebacterium* spp., *Kocuria* spp., *Bacillus* spp., including *B. cereus*, *Lysinibacillus* spp., *Roseomonas mucosa*, *Aerococcus viridans*, *Dolosigranulum pigrum*, *Pseudomonas oryzihabitans*, *Moraxella osmosis*, *Acinetobacter lwoffii*, *Stenotrophomonas maltophilia*, *Candida parapsilosis*, and *Micrococcus luteus.* In the present investigation, *Candida* spp. were detected in the residual content, on the dropper tip, and on the cap of only 1 eye drop bottle returned by a patient out of 110 collected bottles in phase 1. The literature review showed that numerous studies reported the microbial contamination of eye drop content with *Candida* spp. [8].

These findings highlight the need for an effective antimicrobial technology incorporated into the dropper tip and cap. Therefore, the microbial contamination of eye drops is a complex multifactorial issue that may benefit from a bundle of complementary interventions [8]. These interventions encompass education and awareness for healthcare professionals and patients, clear instructions for use and the implications of hazardous behaviors given to patients, stringent preventive hygiene measures, and the use of a protective antimicrobial tip and cap [8].

The subsequent phase of the study examined the effectiveness of the integrated Pylote technology into the eye drop tip and cap. Pylote antimicrobial technology is a patented breakthrough innovation [88,89] that can be integrated into numerous porous and non-porous surfaces without changing the manufacturing process [90]. The technology consists of mineral oxide microspheres that are non-metal, non-ionic, and non-nanoparticle-based. Pylote SAS technology is non-release, non-leaching, and friendly to humans. The mineral microspheres are high-purity ceramic particles, including zinc or magnesium oxides, manufactured via a one-step proprietary cleantech process called Pyrolyse Pulvérisée^TM^ [91]. ZnO and MgO are authorized additives for parenteral pharmaceutical containers according to the *European Pharmacopoeia*, *United States Pharmacopeia*, and *Japanese Pharmacopeia* [91]. The mechanism of action is not photo-activation and depends on microsphere surface defects called oxygen vacancies [92]. The mineral microspheres are electron donors that generate, when in contact with water or oxygen, reactive oxygen species (ROS), mainly hydroxyl radicals [93]. These compounds with oxidative properties are rapidly biodegradable active molecules [94,95,96]. The oxidizing radicals produced at the microsphere surface lead, by direct contact, to the destruction of a broad spectrum of GPB and GNB, resistant bacteria, in addition to viruses (including those causing conjunctivitis), and have a lesser effect on fungi [90,91]. The potent antimicrobial activity of microspheres is due to the sphericity coefficient of ≥0.75, characterized by a narrow distribution size and even distribution on integrated surfaces, where the determined distance between two microspheres is between 0.2 and 1 µm [91]. The antimicrobial effect occurs in nanoseconds within a few nanometers of the incorporated surface without the release or consumption of the microspheres [91]. The antimicrobial activity of Pylote mineral microspheres is dose- and time-dependent [91]. Pylote technology has demonstrated high effectiveness in vitro, in vivo, and in situ and has shown sustainable effects under real-life and worst-scenario conditions. Previous tests [90] were undertaken according to the ISO 22196:2011, JIS Z 2801, ISO 20743:2021, and NF S90-700:2019 standards.

In the present study, Activated RispharmTM eye drop containers, integrating Pylote technology into the dropper tips and inner caps, were tested against standard plastic containers. The inoculated bacteria and fungi were selected according to the literature review [8] and the recommendations of the *European Pharmacopoeia* for testing antiseptic medicinal products [34]. Pylote technology has demonstrated effective antimicrobial activity in vitro against a wide range of microorganisms, including *S. aureus* (NCTC 10788), *P. aeruginosa* (NCTC 12924), and, to a lesser extent, *C. albicans* (NCPF 3179) [91]. The inoculated microbial load of each microorganism was similar to that in the tests previously conducted. At baseline, standard eye drops collected from the patients and the hospital showed unlimited microbial growth of the two tested bacteria at the dropper tip and cap sites. The second phase showed that Activated Rispharm^TM^ packaging inoculated with *S. aureus* and *P. aeruginosa* exhibited more than one log reduction compared with the control, while the standard packaging showed unlimited growth. Although Pylote technology showed a higher reduction in microbial load in in vitro studies [91], these differences can be related to sampling, swabbing, the methodology of microbiological culture in vitro, and the surface type cultured (flat coupons versus dropper tips and caps). The methods applied in the in vitro study [91] consisted of recovering the microorganisms by dispersion on the plates, dilution, and culture in broth cultures, compared with the swabbing method used in this experiment.

For *C. albicans*, there was no observed difference between the different types of packaging. A reduction of about 1 log was observed between the control and the packaging.

The Pylote technology maintained a low level of contamination with the microorganisms. In this experiment, dilution was not undertaken, and although it may not have affected the reliability of the results, it could have further demonstrated the antimicrobial efficiency of the technology in preventing the contamination of the incorporated surfaces.

A thirty-year literature review of dropper tip and cap microbial contamination found that *Staphylococcus* spp. are the predominant microorganisms isolated from in-use eye drops [8]. In the present experiment, the results showed the high effectiveness of Pylote technology in reducing *S. aureus* microbial load, while its effect on *P. aeruginosa* was more limited compared to the control. Of note, standard packaging showed high microbial proliferation in the same conditions. The tested in-use eye drops in this study did not show any contamination with *P. aeruginosa*. Additionally, the methods of swabbing and culturing in vitro differed, although the protocol used by Pylote’s in vitro studies was compatible with international standard requirements [91].

The final phase examined the feasibility of integrating Activated Rispharm^TM^ packaging into eye drop production at the Pharmacy Unit—UPSO2—and the practical applicability during use. Activated Rispharm^TM^ packaging appears well suited for the effective instillation of eye drops. The absence of malfunction during the production and installation process of eye drops from Activated Rispharm^TM^ packaging suggests the feasibility of larger-scale production.

## 5. Conclusions

The microbial contamination of the dropper tip and inner cap surfaces of in-use eye drops and the capacity to control these risks using innovative patented technology were demonstrated in a two-phase study conducted at the Pharmacy Unit—UPSO2. The results highlighted the proven but often-disregarded microbial contamination of these surfaces (tip and cap) and their contribution to cross-contamination and increased risk of infection, particularly for susceptible users. Microbial contamination is a multifaceted problem necessitating multiple parallel interventions. In their pursuit of patient protection and continuous improvement, the Pharmacy Unit evaluated the unique green technology by Pylote SAS to examine its effectiveness, applicability, and feasibility for integration into the manufacturing process. The results showed promising effects that combine effectiveness, safety, and sustainability to protect the patient by preventing infection risk due to the contamination of eye drop containers.

## Figures and Tables

**Table 1:** Microbial loads on Activated Rispharm™ packaging and standard packaging after 24 h of contact (mean ± standard deviation).

Microbial Load (CFU)	*Staphylococcus aureus*	*Pseudomonas aeruginosa*	*Candida albicans*
At C0/mL)	**5.2 × 10^5^**	**1.7 × 10^5^**	**2.4 × 10^3^**
After C24 h in 0.9% NaCl (/mL)	**4.3 × 10^3^**	**8.0 × 10^1^**	**2.2 × 10^3^**
After A24h contact with standard eye drop packaging (cap + tip)	**>3.3 × 10^3^**	**>3.3 × 10^3^**	**3.0 × 10^2^** **±** **0.4 × 10^2^ CFU**
**After 24 h contact with Activated RispharmTM eye drop packaging (cap + tip)**	**2.1 × 10^2^** **±** **7.0 × 10^1^**	**3.2 × 10^2^** **±** **9.0 × 10^1^**	**2.4 ×10^2^** **±** **6.0 × 10^1^**
Log reduction Pylote TM vs. standard eye drop packaging	**>1.2**	**>1.1**	0 *

C0: at 0 hour for control; C24h: after 24 hours for control; A24h: after 24 hours for assay; * Value considered 0, but lower for Activated Rispharm^TM^ packaging than for the standard one. The average is the mean average between two tested times.

## Data Availability

All data was included in the article.

## Appendix A

**Table A1:** Validation of sampling and culture methods.

	*S. aureus*		*P. aeruginosa*		*C. albicans*	
	Inoculated number at 0 h	Detected number after 24 h contact time	Inoculated number at 0 h	Detected number after 24 h contact time	Inoculated number at 0 h	Detected number after 24 h contact time
**Bottle 1**	10^3^ CFU	6.5 × 10^2^ CFU	10^3^ CFU	1.1 × 10^2^ CFU	10^2^ CFU	1.7 × 10^1^ CFU
**Bottle 2**		4.6 × 10^2^ CFU		3.6 × 10^2^ CFU		2.2 × 10^1^ CFU
**Bottle 3**		5.6 × 10^2^ CFU		2.0 × 10^2^ CFU		1.6 × 10^1^ CFU
**Mean ± SD**		5.6 × 10^2^ CFU ± 9.55 × 10^1^ CFU		2.2 × 10^2^ CFU ± 1.3 × 10^2^ CFU		1.8 × 10^1^ CFU ± 0.3 × 10^1^ CFU
